# Evaluating the Long-Term Health and Economic Impacts of Central Residential Air Filtration for Reducing Premature Mortality Associated with Indoor Fine Particulate Matter (PM_2.5_) of Outdoor Origin

**DOI:** 10.3390/ijerph120708448

**Published:** 2015-07-21

**Authors:** Dan Zhao, Parham Azimi, Brent Stephens

**Affiliations:** Department of Civil, Architectural and Environmental Engineering, Illinois Institute of Technology, Chicago, IL 60616, USA; E-Mails: dzhao14@hawk.iit.edu (D.Z.); pazimi@hawk.iit.edu (P.A.)

**Keywords:** indoor air, HVAC filter, DALYs, premature mortality, exposure, infiltration

## Abstract

Much of human exposure to fine particulate matter (PM_2.5_) of outdoor origin occurs in residences. High-efficiency particle air filtration in central heating, ventilating, and air-conditioning (HVAC) systems is increasingly being used to reduce concentrations of particulate matter inside homes. However, questions remain about the effectiveness of filtration for reducing exposures to PM_2.5_ of outdoor origin and adverse health outcomes. Here we integrate epidemiology functions and mass balance modeling to estimate the long-term health and economic impacts of HVAC filtration for reducing premature mortality associated with indoor PM_2.5_ of outdoor origin in residences. We evaluate 11 classifications of filters (MERV 5 through HEPA) using six case studies of single-family home vintages and ventilation system combinations located in 22 U.S. cities. We estimate that widespread use of higher efficiency filters would reduce premature mortality by 0.002–2.5% and increase life expectancy by 0.02–1.6 months, yielding annual monetary benefits ranging from $1 to $1348 per person in the homes and locations modeled herein. Large differences in the magnitude of health and economic impacts are driven largely by differences in rated filter efficiency and building and ventilation system characteristics that govern particle infiltration and persistence, with smaller influences attributable to geographic location.

## 1. Introduction

Elevated outdoor concentrations of fine particulate matter (*i.e.*, PM_2.5_, or the mass concentration of particles less than 2.5 μm) are consistently associated with a number of adverse health effects ranging from increased lung cancer to mortality [1,2,3,4,5,6]. However, because outdoor particles infiltrate into buildings where people spend most of their time [7], much of human exposure to PM_2.5_ of outdoor origin actually occurs indoors, and in particular, inside residences [8,9,10,11,12,13,14,15,16,17,18,19,20,21,22]. High-efficiency particle air filtration in central heating, ventilating, and air-conditioning (HVAC) systems is increasingly being used to reduce concentrations of particulate matter of both indoor and outdoor origin inside residences [23,24,25,26,27,28,29,30,31,32]. Several recent studies have also demonstrated that higher-efficiency central HVAC particle air filtration in buildings such as schools, offices, retail stores, and residences is likely to have large benefits for reducing chronic health effects and monetary costs associated with exposure to particulate matter [33,34,35,36]. However, few studies have evaluated the impacts of HVAC filtration on chronic health effects of PM_2.5_ of outdoor origin inside residences (e.g., [36]), and they have been somewhat limited in their exploration of the influence of several key assumptions.

Therefore, in this work, we integrate epidemiology functions and mass balance modeling to estimate the long-term health and economic impacts of particulate matter air pollution and tailor them for use in evaluating control strategies in residential indoor environments. We then estimate the long-term health and economic impacts of central HVAC filtration for reducing premature mortality associated with indoor PM_2.5_ of outdoor origin in residences. We evaluate a wide range of 11 classifications of HVAC filters ranging from a minimum efficiency reporting value of 5 (MERV 5 [37]) to high-efficiency particulate arrestance (HEPA) filters using six case studies of single-family home vintages and ventilation system combinations (including older, existing, and new homes, both with and without mechanical ventilation systems) located in 22 U.S. cities. We also explore the use of a novel alternative approach for predicting mortality impacts of HVAC filters: increased life expectancy. We specifically explore the influence of key input parameters including (1) concentration–response functions for estimating premature mortality and life expectancy outcomes; (2) geographic location and annual average outdoor PM_2.5_ concentrations; (3) building characteristics such as envelope airtightness, envelope penetration factors, and HVAC system runtimes; and (4) ventilation strategies including infiltration-only and three common mechanical ventilation system types designed to meet ASHRAE Standard 62.2 [38]: constant exhaust-only, constant supply-only, and central-fan-integrated-supply (CFIS) with constant exhaust. 

## 2. Methods

The following sections detail the methods we used to estimate the long-term health and economic impacts of central HVAC filtration for reducing premature mortality (and increasing life expectancy) associated with indoor PM_2.5_ of outdoor origin in the selected case study residences using common epidemiology functions (Section 2.1) and a set of time-averaged well-mixed indoor air pollutant mass balance models (Section 2.2).

### 2.1. Health and Economic Impact Modeling Methods

In Section 2.1, we describe the methods and ranges of input parameters we used to evaluate the health and economic impacts of HVAC filtration in the case study homes.

#### 2.1.1. Estimating Health and Economic Endpoints Associated with Air Pollution

There are several endpoints commonly used to evaluate the health and economic impacts associated with chronic exposure to air pollution, including mortality [34], life expectancy [1,39], disability-adjusted life-years (DALYs) due to a variety of outcomes such as mortality, chronic bronchitis, nonfatal stroke, hospital admissions, and others for PM_2.5_ [33,35,36,40], and the monetary value of increasing or avoiding adverse health effects [41]. Some of these endpoints have been evaluated only for outdoor air pollutants. Others have been adapted and modified to account for indoor exposures to pollutants of either indoor or outdoor origin. Further, there are a variety of response functions and associated inputs commonly used for estimating each health endpoint. Most widely adopted methods rely on estimating a change in health endpoint (Δ*y_i_*) due to a change in pollutant concentration based on a concentration–response (C-R) function derived from epidemiology studies [5,33,40,42], typically following a variation of the generic form shown in Equation (1) [43].

(1) $$∆y_{i}=y_{0}\left[\text{exp}\left(\beta_{i}\times ∆x_{i}\right)-1\right]$$

where:

Δy_i_ = change in annual health endpoint (per person per year)
*y*_0_ = annual baseline prevalence of illness (per person per year)
*β_i_* = C-R endpoint effect estimate for pollutant *i* (e.g., per μg/m^3^ of pollutant *i*)
Δ*x_i_* = change in concentration or exposure (e.g., μg/m^3^ of pollutant *i*)

Once a change in a specific health endpoint such as mortality is known, other metrics such as DALYs or monetary benefits are readily estimated. A change in DALYs is commonly estimated using Equation (2) [44,45,46,47,48].

(2) $$DALYs=\;\frac{\partial DALYs}{\partial disease\;incidence}\times ∆y_{i}$$

where:

*DALYs* = disability-adjusted life-years associated with change in health endpoint (per person per year)
$\frac{\partial DALYs}{\partial disease\;incidence}$ = DALYs lost per incidence

Similarly, the economic impacts (*A_i_*) of various health endpoints such as DALYs or premature mortality are readily estimated by Equation (3).

(3) $$A_{i}=\;∆y_{i}\times {\$}_{i}$$

where:

*A_i_* = value of avoided morbidity or mortality endpoint ($ per person per year)
$*_i_* = monetary value per incident ($/incident)

Clearly, the selection of C-R functions and values for *βi*, Δ*xi*, $\frac{\partial DALYs}{\partial disease\;incidence}$, and $*_i_* greatly influence outcomes of evaluations of the health and economic impacts of indoor or outdoor air pollution. Next, we review each of these inputs from the existing literature with a specific focus on the long-term mortality-related health impacts of outdoor PM_2.5_ in the U.S. and make slight modifications to tailor them for application in evaluating control strategies such as HVAC filtration in residential indoor environments.

#### 2.1.2. C-R Endpoint Effect Estimates for Long-Term PM_2.5_ Mortality

A wide variety of C-R endpoint effect estimates (*i.e.*, *β_i_* from Equation (1)) have been derived in epidemiology studies that explored associations between outdoor concentrations of PM_2.5_ (and other pollutants) and adverse health outcomes such as premature mortality, hospital admissions, asthma-related emergency room visits, and others. Many of these effect estimates are reviewed in the U.S. Environmental Protection Agency (EPA) report *The Benefits and Costs of the Clean Air Act from 1990 to 2020* [41] and other supporting documents [43]. Central estimates of *β_i_* for increases in premature mortality associated with long-term outdoor PM_2.5_ concentrations in the U.S. commonly range from 5.8% per 10 µg/m^3^ (*i.e.*, 0.58% per µg/m^3^, derived from the American Cancer Society cohort [3]) to 15% per 10 µg/m^3^ (*i.e.*, 1.5% per µg/m^3^, derived from a re-analysis of the Harvard Six Cities cohort [49]). Other common values for *β_i_* for premature mortality associated with outdoor PM_2.5_ in the U.S. include 1.26% per µg/m^3^ from the original Six Cities analysis [5], 1.17% per µg/m^3^ from another large cohort [42], and midpoint values or more subjective values such as 1.06% per µg/m^3^ based on expert panel solicitations [43,50]. Although some epidemiology studies have also found large geographic differences in C-R functions due to factors such as varying PM_2.5_ composition, cohort exposure, or population susceptibility characteristics [51,52,53], it is still common to assume that all PM_2.5_ is equally potent in producing premature mortality regardless of chemical composition [41].

#### 2.1.3. Response Functions for Indoor PM_2.5_ of Outdoor Origin

While the general C-R function described in Section 2.1.1 and values for *β_i_* described in Section 2.1.2 were originally derived for outdoor concentrations of PM_2.5_ and other pollutants, several recent studies have also modified them for use in indoor environments. Logue *et al.* (2012) [40] first proposed a modified version of Equation (1) to estimate a variety of chronic health endpoints (including premature mortality) from indoor exposures to a variety of pollutants of both indoor and outdoor origin (including PM_2.5_), as shown in Equation (4) [40]. Note that Equation (4) can be used broadly for multiple pollutants and health endpoints, although here we focus solely on premature mortality associated with indoor PM_2.5_ of outdoor origin. Equation (4) can also be modified to present a relative change in a health endpoint for a given change in Δ*C_in,exposure_* (*i.e.*, Δ*y_i_*/*y*_0_) that is independent of the existing baseline prevalence of illness.

(4) $$∆y_{i}=-y_{0}\left[\text{exp}\left(-\beta_{i,indoor}\times ∆C_{in,exposure}\right)-1\right]$$

where:

*β_i,indoor_* = C-R endpoint effect estimate for a change in long-term indoor PM_2.5_ concentration (per μg/m^3^)
Δ*C_in,exposure_* = change in indoor PM_2.5_ exposure concentration (μg/m^3^)

Logue *et al.* (2012) [40] used a central estimate of *β_i,indoor_* = 0.58% per µg/m^3^ for premature mortality associated with both indoor and outdoor origin PM_2.5_, which was taken directly from the outdoor epidemiology literature [3]. Other recent studies have used similar methods to estimate health and economic impacts of indoor exposures to PM_2.5_ and other pollutants, albeit often with different assumed values for *β_i,indoor_* [33,35,36,54] (including those for PM_2.5_ reviewed in Section 2.1.2). 

Further, Logue *et al.* (2012) [40] also considered an important adjustment to Δ*C_i,in,exposure_* in Equation (4) to account for the amount of time the average American spends inside their home (about 70% [7]), as shown in Equation (5.)

(5) $$∆C_{in,exposure}=∆C_{in}\times P_{time,indoor}$$

where:

Δ*C_in_* = change in indoor PM_2.5_ concentration (µg/m^3^)
*P_time,indoor_* = fraction of time spend inside a residence (0.7)

Since this method has been used successfully in a number of recent studies for evaluating the health and economic impacts of indoor air pollution, including PM_2.5_ [33,35,40,54], it serves as the basis for our health and economic modeling herein, albeit with some key modifications as described in the next sections.

#### 2.1.4. Modified Response Functions for Indoor PM_2.5_ of Outdoor Origin

One limitation to the aforementioned efforts to model health outcomes of indoor air pollution is the use of a C-R endpoint estimate (*β_i,indoor_*) that is taken directly from the epidemiology literature on outdoor air pollution (*i.e.*, assuming that *β_i,indoor_* = *β_i_*). Because people spend the majority of their time indoors, there is actually an underlying change in indoor concentrations (and thus indoor exposures) associated with any change in outdoor concentrations that were previously observed in epidemiology studies. In order to model the health impacts of using a control strategy such as HVAC filtration to reduce indoor proportions of outdoor PM_2.5_, we consider it more appropriate to adjust the original derived endpoint effects from the outdoor air pollution literature to account for the underlying infiltration into residential indoor environments that would have necessarily occurred during the observation period. This allows us to generate a modified C-R endpoint estimate to account for the equivalent changes in indoor PM_2.5_ concentrations that would occur as a consequence of changes in outdoor PM_2.5_ concentrations, which we then use to directly link changes in indoor PM_2.5_ concentrations (which are modeled in Section 2.2) to changes in health endpoints. Several recent studies demonstrate the importance and utility of similar modifications for improving the accuracy and representativeness of C-R response functions and endpoint effect estimates for PM_2.5_ [22] as well as PM_10_ [55] and ozone [56]. However, these same efforts to account for outdoor pollutant infiltration have not been merged with the recent health endpoint modeling efforts based on Equations (1) and (4) [33,35,40]. 

Therefore, we have integrated these methods and adjusted the previously derived endpoint effect estimates for premature mortality associated with outdoor PM_2.5_ concentrations from the literature (*β_i_*) to estimate an underlying value of *β_i,indoor_* that takes into account the inherent changes in indoor exposures to PM_2.5_ of outdoor origin that would have occurred in the same epidemiology investigations that relied on outdoor PM_2.5_ concentrations alone. This is accomplished by assuming a population-average residential outdoor PM_2.5_ infiltration factor of across U.S. residences, as shown in Equation (6). This modification is similar in concept to other regression calibration methods used in the epidemiology literature [57].

(6) $$\beta_{i,indoor}=\frac{\beta_{i}}{F_{inf}}$$

where:

$\beta_{i}$ = endpoint effect estimate for premature mortality associated with outdoor PM_2.5_ (% per μg/m^3^)
*F_inf_* = population-average PM_2.5_ infiltration factor in U.S. residences

As an example of this modification, let us assume a PM_2.5_ endpoint effect (*β_i_*) for premature mortality of 10.6% per 10 μg/m^3^ and that the population-average PM_2.5_ infiltration factor in U.S. residences is 0.6 [8]. If the long-term average outdoor PM_2.5_ concentration decreased by 10 μg/m^3^ during the time period of the original investigation, the corresponding underlying decrease in the average indoor PM_2.5_ concentration would have been only 6 μg/m^3^, while the observed changes in health endpoints would not have changed (*i.e.*, premature mortality was still reduced by 10.6%). Therefore, we can generate a new endpoint effect estimate for premature mortality associated with long-term indoor exposures to PM_2.5_ that infiltrates from outdoors (*i.e.*, *β_i,indoor_* from Equation (6)). We can then use that value with Equations (4) and (5) to predict the influence of changing infiltration factors (*i.e.*, Δ*C_in_*) due to control strategies such as HVAC filtration on mortality outcomes associated with outdoor PM_2.5_. We should note that underlying values of *F_inf_* for PM_2.5_ actually vary among homes both within and between geographic regions due to varying home characteristics [16,17,18,55], but it is beyond the scope of this work to attempt to re-assign exposure estimates to individuals in previous epidemiology studies from which *β_i_* values were originally derived. While this should be explored in future work, similar to Chen *et al.* (2012) for PM_10_ [55], we consider it appropriate at this time to use only population average estimates of the underlying infiltration of outdoor PM_2.5_ to estimate *β_i,indoor_*.

#### 2.1.5. An Alternative Approach: The Impact of Indoor PM_2.5_ of Outdoor Origin on Life Expectancy

Finally, in a separate effort, we also explore the use of an alternative approach for predicting mortality impacts of HVAC filters: the impact on increased life expectancy. In a seminal study of the impacts of long-term changes in outdoor PM_2.5_ concentrations, Pope *et al.* (2009) demonstrated that a 10 μg/m^3^ decrease in long-term ambient PM_2.5_ concentrations across 51 U.S. metropolitan areas from 1980 to 2000 led to a mean (± SE) increase in life expectancy of 0.61 ± 0.20 years [1]. This work provides another important health endpoint associated with PM_2.5_ of outdoor origin that, to our knowledge, has not been used to study indoor environments. However, the reported linear relationship must again be modified to account for the underlying changes in indoor exposures to outdoor-infiltrated PM_2.5_ that would have occurred in the original study. Doing so allows for estimating the equivalent changes in indoor PM_2.5_ concentrations that would occur as a consequence of changes in outdoor PM_2.5_ concentrations. Therefore, we again modified this relationship to account for population-average residential PM_2.5_ infiltration factors (*F_inf_* = 0.6) using Equation (7).

(7) $${\left(\frac{\text{Δ}LE}{\text{Δ}C}\right)}_{indoor}=\frac{{\left(\frac{\text{Δ}LE}{\text{Δ}C}\right)}_{outdoor}}{F_{inf}}$$

where:

${\left(\frac{\text{Δ}LE}{\text{Δ}C}\right)}_{outdoor}$ = life expectancy factor associated with changes in outdoor PM_2.5_ concentration (years per μg/m^3^)
${\left(\frac{\text{Δ}LE}{\text{Δ}C}\right)}_{indoor}$ = modified life expectancy factor associated with changes in indoor PM_2.5_ of outdoor origin (years per μg/m^3^)

Changes in life expectancy attributable to changes in outdoor PM_2.5_ infiltration are then estimated by multiplying the life expectancy factor modified for indoor environments (Equation (7)) by a predicted change in indoor PM_2.5_ exposures (Equation (4)), as shown in Equation (8).

(8) $$∆LE={\left(\frac{\text{Δ}LE}{\text{Δ}C}\right)}_{indoor}∆C_{in,exposure}$$

where:

Δ*LE* = estimated change in life expectancy due to a change in indoor exposures (years)

We should note that this life expectancy modeling effort is conducted independently from the premature mortality modeling efforts and that the two outcomes are essentially different measures of the same mortality-related endpoints. Estimates of changes in life expectancy are primarily intended to explore the utility of this alternative approach and provide a novel method for estimating mortality outcomes that may be more readily understood by the general public. Results from the two efforts should not be combined, otherwise mortality impacts would be double-counted.

#### 2.1.6. Health and Economic Modeling Inputs Used in This Work

After reviewing and integrating the combinations of methods and key parameters described above, we selected reasonable values for model inputs in order to evaluate the long-term mortality-related health and economic impacts associated with changes in indoor exposures to PM_2.5_ of outdoor origin inside residences due to improved central HVAC filtration. Table 1 summarizes all input parameters used for the health and economic impact modeling in this work. All values for changes in indoor PM_2.5_ concentrations (Δ*C_in_*) fro the case study homes are modeled using the mass balance modeling methods described in Section 2.2.

We used the C-R function in Equation (4) and the same central estimate of *β_i_* used by the U.S. EPA (1.06% per µg/m^3^) [41], along with a population-average *F_inf_* for PM_2.5_ of 0.6 [8] to establish consistent values of *β_i,indoor_* derived from the epidemiology literature and modified to account for infiltration into the indoor environment. We also explore the influence of lower and upper bounds of *β_i_* of 0.58% per µg/m^3^ and 1.5% per µg/m^3^, respectively, from the work reviewed in Section 2.1.2. Values for $\frac{\partial DALYs}{\partial disease\;incidence}$ for chronic PM_2.5_ health endpoints commonly include 1.2 for chronic bronchitis, 1.4 for mortality, and 13.1 for nonfatal strokes with complications [40]. Limiting to the total DALYs lost due to premature mortality alone, we used the same values reported in Logue *et al*. (2012) [40]: 1.4 DALYs lost per incidence. We should note that although Logue *et al.* (2012) [40] also report this value within a 95% confidence interval (CI) of 0.14 to 14 per incidence, we do not perform a systematic investigation of the sensitivity of our results to this range, primarily for simplicity and clarity (put simply: an order of magnitude difference in DALYs lost per incidence necessarily yields an order of magnitude difference in both total DALYs lost and the associated monetary value). 

Since DALYs are estimated based on existing baseline rates of premature mortality (*y*_0_), we obtained *y*_0_ values over the four most recent years for which data are available from the National Vital Statistics System (NVSS) (2010–2013) [58]. Reported values for annual baseline rates of premature mortality are 7.46 × 10^–3^, 7.40 × 10^-3^, 7.32 × 10^-3^, and 7.32 × 10^-3^ per person for years 2010 to 2013 respectively. We used an arithmetic mean of these most recent values: 7.38 × 10^-3^ (*i.e.*, 738 per 100,000). This value represents the average age-adjusted death rate over the past four years, which accounts for changes in the age distribution of the population and is considered representative of the entire population. Specific population demographics are not considered herein.

The monetary value per incident ($*_i_*) is commonly based on the concept of the value of statistical life (VSL). In the U.S., three main governmental organizations utilize this metric in their economic analyses, including the U.S. EPA, the Food and Drug Administration (FDA), and the Department of Transportation (DOT). EPA recommends that a central estimate of $7.4 million (in $2006), updated to the year of the analysis, be used in all benefits analyses that seek to quantify mortality risk reduction benefits [59]. For premature mortality, the FDA uses a VSL estimate of $5 million, without specifying a dollar year, and occasionally provides alternative estimates using higher or lower values [60]. DOT suggested a VSL of $9.1 million in current dollars in 2013 and $9.2 million in 2014 [61]. For this work, we used the arithmetic mean of the latest reported VSL values from these three organizations (*i.e.*, $7.2 million) to estimate the economic impacts of premature mortality associated with PM_2.5_ exposure. Again we consider these inputs and outputs on a per-person basis using national average statistics and do not consider the influence of specific population demographics.

Finally, for estimating changes in life expectancy, we used a range of values for ${\left(\frac{\text{Δ}LE}{\text{Δ}C}\right)}_{outdoor}$ reported in the literature, including 0.61 ± 0.20 years per 10 µg/m^3^ reported by Pope *et al*. (2009) [1], as well as 0.56 ± 0.19 and 0.35 ± 0.16 years per 10 µg/m^3^ reported in a reanalysis of the same data by Correia *et al.* (2013) for the time periods spanning 1980–2007 and 2000–2007, respectively [62]. We used the arithmetic mean of these three quantities as our central estimate (*i.e.*, 0.51 years per 10 µg/m^3^ or 0.051 years per µg/m^3^), but also tested the sensitivity to lower and upper bounds using their reported uncertainty values (*i.e.*, 0.019 and 0.081 years per µg/m^3^, respectively). We did not attempt to estimate the economic value of increasing life expectancy, although a “value per statistical life-year” (VSLY) approach could be utilized with more specific knowledge of population demographics for a particular model scenario (e.g., [34]). 

**Table 1:** Summary of input parameters for the health and economic impact modeling.

Modeling outputs	Inputs		Mean	Range	Ref.
*Change in annual health endpoint (Δy_i_/y_0_)*	C-R endpoint effect estimate for a change in long-term outdoor PM_2.5_ concentrations	*β_i_*	1.06% (per µg/m^3^)	0.58–1.5% (per µg/m^3^)	[41], [3], [49]
	Population-average infiltration factor for PM_2.5_	*F_inf_*	0.6	N/A	[8]
	Fraction of time spent inside a residence	*P_time,indoor_*	70%	N/A	[7]
	Annual baseline prevalence of illness	*y_0_*	7.38 × 10^–3^ (per person per year)	N/A	[58]
*Change in DALYs*	DALYs lost per incidence	$\frac{\partial DALYs}{\partial disease\;incidence}$	1.4	N/A	[40]
*Change in value of avoided mortality endpoint (ΔA_i_)*	Monetary value per incident	$_i_	$7.2 million	N/A	[59], [60], [61]
*Change in life expectancy (ΔLE)*	Life expectancy factor associated with changes in outdoor PM_2.5_ concentration	${\left(\frac{\Delta LE}{\Delta C}\right)}_{outdoor}$	0.051 (years per µg/m^3^)	0.019–0.081 (years per µg/m^3^)	[1], [62]

In the next section, we describe methods used to estimate the final key input parameter for the health and economic impact modeling, Δ*C_in_*, using mass balance models applied to several case study homes. 

### 2.2. Modeling Indoor Concentrations of Outdoor PM_2.5_

We combined the methods described in Section 2.1 with mass balance models and applied them to a suite of case study homes with widely varying building characteristics in multiple geographic locations. We used a simple time-averaged well-mixed mass balance to estimate the long-term annual average indoor concentration of PM_2.5_ of outdoor origin in each scenario in each location [63]. We did not include any indoor sources or resuspension activities in any of the scenarios. Averages of each input parameter were gathered as follows: values for outdoor PM_2.5_ concentrations were gathered from local air quality monitoring stations in each chosen location; values for envelope penetration factors, deposition loss rate constants, HVAC filtration efficiency, and HVAC airflow rates and recirculation rates were culled from the literature; and values for air exchange rates and HVAC system runtimes were modeled using a building energy simulation program (BEopt combined with EnergyPlus). The full, integrated modeling process (both mass balance and health and economic impact modeling) is described in Figure 2.

#### 2.2.1. Model Home Selection

We used three primary vintages of single-family residences located in 22 U.S. cities as individual case studies, including (1) older, (2) existing, and (3) new homes. The home types were defined largely by their envelope airtightness and insulation characteristics as well as their heating and cooling system characteristics. The three home types were chosen to reflect wide variations in envelope air tightness, envelope PM_2.5_ penetration, and HVAC system runtimes that are common across home vintages. Each home was a single-story detached single-family home with the same basic geometry, with three bedrooms, two bathrooms, 188 m^2^ of floor area, a volume of 459 m^3^, a natural gas furnace, and a central forced-air air conditioning system. Full descriptions of the model homes are provided in Azimi *et al.* (2015) [64]. 

Briefly, the new homes were chosen to represent modern high-efficiency homes with well-insulated building envelopes (meeting modern code requirements), high airtightness (3 ACH_50_ in all climate zones per IECC 2012 [65]), and properly sized high efficiency heating and air-conditioning systems for each climate zone. The existing homes were chosen to represent typical older and less efficient homes with moderate outdoor particle infiltration by incorporating moderately insulated building envelopes, typical airtightness (10 ACH_50_), and larger and less efficient heating and air-conditioning systems for each climate zone based on typical existing home characteristics in each area. Finally, the older homes were chosen to represent typical older vintage homes with high outdoor particle infiltration by incorporating poorly insulated building envelopes, low airtightness (20 ACH_50_), and larger and less efficient (and often undersized) heating and air-conditioning systems for each climate zone based on typical older vintage home characteristics in each area. 

Using the same base model home geometry for each home type, we explored six combinations of home vintage and ventilation strategy, including three infiltration-only scenarios and three mechanical ventilation scenarios. For the infiltration-only scenarios, we considered (1) older, (2) existing, and (3) new homes relying on infiltration alone (*i.e.*, no mechanical ventilation other than intermittent bathroom and kitchen exhaust). For the three mechanical ventilation scenarios, we considered only the new home vintage and assumed it was designed to meet ASHRAE Standard 62.2 [38] using three common types of mechanical ventilation systems: (1) constant exhaust-only, (2) constant supply-only, and (3) central-fan-integrated-supply (CFIS) with constant exhaust. We did not consider window-opening or door-opening behaviors in any of the scenarios. 

#### 2.2.2. Mass Balance Modeling

For the three infiltration-only scenarios, long-term average indoor concentrations of PM_2.5_ of outdoor origin were estimated using Equation (9).

(9) $$C_{in}=F_{inf}C_{out}=\frac{P\lambda_{inf}}{\lambda_{inf}+k_{dep}+\left(f\eta_{HVAC}\lambda_{HVAC}\right)}C_{out}$$

where:

*F_inf_* = PM_2.5_ infiltration factor for homes without mechanical ventilation (-)
*P* = PM_2.5_ penetration factor of the building envelope (-)
*λ_inf_* = air exchange rate due to infiltration (h^–1^)
*C_out_* = outdoor PM_2.5_ concentration (μg/m^3^)
*k_dep_* = first-order indoor PM_2.5_ deposition loss rate coefficient (h^–1^)
*η_HVAC_* = PM_2.5_ removal efficiency of the HVAC filter (-)
*λ_HVAC_* = HVAC system recirculation rate (HVAC airflow rate divided by volume, h^–1^)
*f* = fractional operation time of the HVAC system (-)

For the three mechanical ventilation scenarios, we modeled only the new home vintage, as it was the only vintage that would likely be built to meet ASHRAE Standard 62.2 using mechanical ventilation systems. Three mechanical ventilation scenarios were evaluated, including:

1. Constant exhaust-only ventilation;
2. Constant supply-only ventilation; and
3. Central-fan-integrated-supply (CFIS) with constant exhaust.

These common system types are described in detail in Walker and Sherman (2013) [66]. The minimum continuous mechanical ventilation airflow rate required was calculated using Equation (10) based on ASHRAE 62.2 Standard [38]. The minimum mechanical ventilation airflow rate for all of the model homes used herein was thus assumed to be 50 cfm (85 m^3^/h). This yields a minimum ventilation air exchange rate of 0.18/h.

(10) $$Q_{fan}=0.01\;A_{floor}+7.5\left(N_{br}+1\right)$$

where:

*Q_fan_* = minimum mechanical ventilation flow rate (cfm)
*A_floor_* = floor area (2025 ft^2^)
*N_br_* = number of bedrooms (3)

In the exhaust-only mechanical ventilation approach, a small exhaust fan was assumed to operate 100% of the time with a constant airflow rate of 50 cfm (85 m^3^/h). Make-up air was assumed to be provided by infiltration through the building envelope. These values were then used in a modified version of the simplified time-averaged infiltration factor model in Equation (9) to predict the annual-average indoor PM_2.5_ concentration of outdoor origin for new homes in each location with exhaust-only ventilation systems, as shown in Equation (11). Because the supply air was assumed to infiltrate through the building envelope with exhaust-only ventilation systems, we assumed that PM_2.5_ penetration factors were the same as the penetration factors for the new homes without mechanical ventilation systems.

(11) $$C_{in}=C_{out}{\text{F}}_{inf,exhaust}=\frac{P\lambda_{total,exhaust}}{\lambda_{total,exhaust}+k_{dep}+\left(f_{HVAC,exhaust}\eta_{HVAC}\lambda_{HVAC}\right)}C_{out}$$

where:

*F_inf,exhaust_* = PM_2.5_ infiltration factor for homes with exhaust-only ventilation systems (-)
*λ_total,exhaust_* = total air exchange rate in new homes with exhaust-only ventilation systems due to a combination of mechanical exhaust and infiltration (h^–1^)
*f_HVAC,exhaust_* = fractional operation time of the HVAC system in new homes with exhaust-only ventilation systems (-)

Similarly, time-averaged indoor PM_2.5_ concentrations of outdoor origin for new homes with supply-only ventilation systems were estimated using Equation (12). In the supply-only ventilation system approach, a small supply fan is assumed to operate 100% of the time with a constant airflow rate of 50 cfm (85 m^3^/h) and outdoor particle penetration is assumed to occur through a combination of intentional mechanical supply and incidental infiltration through the building envelope. In these cases, at least 50 cfm (85 m^3^/h) of outdoor air is supplied directly by the ventilation system and passed through a filter installed inside the small ventilating unit at all times. Any additional air exchange is assumed to occur through infiltration through the building envelope. Therefore, PM_2.5_ penetration factors depend not only on envelope infiltration but also on the assumed removal efficiency of the ventilation system filter. Most manufacturers have not yet adopted high-efficiency filtration systems in small supply ventilator units; therefore, we assume that supply-only mechanical ventilation systems utilize only a MERV 5 filter. Other higher efficiency unit ventilator filtration products are starting to appear on the market, but we are not aware of their widespread use.

(12) $$C_{in}=C_{out}{\text{F}}_{inf,supply}=\frac{\left(1-\eta_{supply}\right)\lambda_{fan}+P\left(\lambda_{total,supply}-\lambda_{fan}\right)}{\lambda_{total,supply}+k_{dep}+\left(f_{HVAC,supply}\eta_{HVAC}\lambda_{HVAC}\right)}C_{out}$$

where:

*F_inf,supply_* = PM_2.5_ infiltration factor for homes with supply-only ventilation system (-)
*λ_fan_* = air exchange rate due to 50 cfm (85 m^3^/h) of supply air provided by the mechanical ventilation system (*i.e.*, 0.18/h)
*λ_total,supply_* = total air exchange rate due to infiltration and ventilation combined (h^–1^)
*f_HVAC,supply_*= fractional operation time of the HVAC system in new homes with supply-only ventilation system (-)
*η_supply_* = PM_2.5_ removal efficiency of MERV 5 supply ventilation system filter (-)

Finally, we also considered a central-fan-integrated-supply (CFIS) system combined with continuous exhaust. In these cases, a 50 cfm (85 m^3^/h) intermittent outdoor air supply is ducted directly into the return plenum of the existing air handling unit and a 50 cfm (85 m^3^/h) exhaust system runs continuously. Therefore, outdoor air enters the indoor environment through a combination of (1) direct supply through the HVAC system and is filtered by the central system filter and (2) infiltration through the building envelope. The relative portion of each airflow path depends on the assumption for HVAC system runtimes, which varied by location because we assumed that the CFIS cycled on and off only to meet heating and cooling needs. In fact, this resulted in the same HVAC system runtimes and total air exchange rates that were modeled in the exhaust-only ventilation scenarios. ASHRAE Standard 62.2 was met in this case based on the 50 cfm (85 m^3^/h) continuous exhaust. Time-averaged PM_2.5_ infiltration factors for the new homes with CFIS systems were estimated using Equation (13).

(13) $$C_{in}=C_{out}{\text{F}}_{inf,CFIS}=\frac{\left(1-f_{HVAC,CFIS}\right)P\lambda_{total,CFIS}+f_{HVAC,CFIS}\left[\left(1-\eta_{HVAC}\right)\lambda_{fan}+P\left(\lambda_{total,CFIS}-\lambda_{fan}\right)\right]}{\lambda_{total,CFIS}+k_{dep}+\left(f_{HVAC,CFIS}\eta_{HVAC}\lambda_{HVAC}\right)}C_{out}$$

where:

*F_inf,CFIS_* = PM_2.5_ infiltration factor for homes with CFIS ventilation systems (-)
*λ_total,CFIS_* = total air exchange rate due to infiltration and ventilation combined in new homes with CFIS ventilation systems (h^–1^)
*f_HVAC,CFIS_* = fractional operation time of the HVAC system in new homes with CFIS ventilation systems (-)

#### 2.2.3. Outdoor PM_2.5_ Concentrations for Each Geographic Location

The selection of cities for modeling was designed to capture all 15 U.S. climate zones in order to yield a wide variation in heating and cooling system operation, as well as the top 15 cities with the highest annual average outdoor PM_2.5_ concentrations summarized in the most recent Integrated Science Assessment for Particulate Matter (data coverage of 2005–2007) [67]. A total of 22 cities were selected for modeling (some of the most polluted cities were in the same climate zone), which are listed below .

1. Boston, MA, 5A12. Blaine (Minneapolis), MN, 6A2. New York, NY, 4A13. Bismarck, ND, 7A3. Philadelphia, PA, 4A14. Colstrip, MT, 6B4. Pittsburgh, PA, 5A15. Pinedale, WY, 7B5. Detroit, MI, 5A16. Denver, CO, 5B6. Atlanta, GA, 3A17. Albuquerque, NM, 4B7. Birmingham, AL, 3A18. Phoenix, AZ, 2B8. St. Louis, MO, 4A19. Riverside, CA, 3B9. Chicago, IL, 5A20. Los Angeles, CA, 3B10. Miami, FL, 1A21. San Francisco, CA, 3C11. Houston, TX, 2A22. Seattle, WA, 4C

Hourly values for outdoor PM_2.5_ concentrations in each of the 22 locations were gathered from the EPA Air Quality System (AQS) for the most recent year data are available (2012) [68]. Missing values were either taken from the nearest monitoring station or linearly interpolated between time periods immediately before and after the missing point. Hourly values were then used to calculate annual average outdoor PM_2.5_ concentrations in each location (*i.e.*, *C_out_*).

#### 2.2.4. HVAC Filtration Efficiency for PM_2.5_

**Figure 1:**
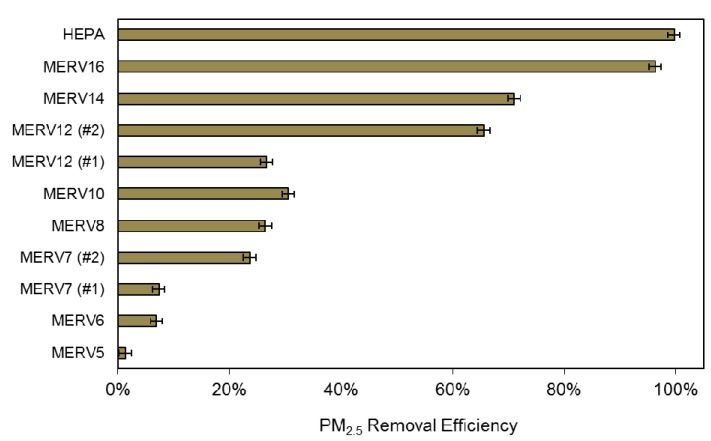
Mean PM_2.5_ removal efficiency for HVAC filters listed by MERV classification.

The most commonly used filtration test standard in the U.S. is ASHRAE Standard 52.2 [37], which evaluates the size-resolved particle removal efficiency of filters for 0.3 to 10 µm particles rather than on a PM_2.5_ mass basis. We recently published a study in which we gathered almost 200 long-term average outdoor particle size distributions measured in locations across the world, mapped them to size-resolved HVAC filtration efficiency for 11 different filters (MERV 5 through HEPA) assuming a slight transformation in size distributions upon penetrating a typical residential building envelope, and estimated their removal efficiency for indoor PM_2.5_ of outdoor origin [69]. We rely on these mean estimates of PM_2.5_ removal efficiencies (*i.e.*, *η_HVAC_*) for 11 MERV classifications including MERV 5, 6, 7, 8, 10, 12, 14, 16, and HEPA, as shown in Figure 1 (data taken directly from Table 2 in [69]). Two different MERV 7 and MERV 12 classified filters are used because they can vary widely in removal efficiency for PM_2.5_ depending on the manufacturer (largely due to their varying performance in the smaller 0.3 to 3 µm size ranges).

#### 2.2.5. PM_2.5_ Penetration Factors and Deposition Loss Rate Coefficients

Next, envelope penetration factors (*p*) and deposition loss rate coefficients (*k_dep_*) were culled from the literature. Envelope penetration factors were assumed to vary according to home vintage [70,71,72]. Values for *P* for PM_2.5_ were taken from the largest study of PM_2.5_ penetration factors in residences of which we are aware, Williams *et al.* (2003) [73], who reported mean *p* = 0.72 across nearly 40 homes, with a minimum of 0.11 and a maximum of 1.0 [73]. We assigned values of 0.11, 0.72, and 1.0 for *p* to the new, existing, and old vintages of homes, respectively. Deposition loss rate coefficients were kept constant for all locations for simplicity. We used the median value for *k_dep_* reported in Wallace *et al.* (2013) [74] (0.70/h), which estimated values based on real-time PM_2.5_ concentrations in over 50 homes in Canada [74]. 

#### 2.2.6. Air Exchange Rates, HVAC Recirculation Rates, and HVAC System Runtimes

Values for annual average HVAC system runtimes (*f*) and air exchange rates (*λ_inf_* or *λ_total_*) were modeled using a whole building energy simulation program: BEopt combined with EnergyPlus. BEopt was used to create the base model geometry and EnergyPlus was used to perform the energy simulations. Simulations were performed using actual meteorological year (AMY) data for the same year (2012) for which outdoor pollutant data were gathered from the AQS. Thermostat set points were held constant in each location at 23.9 °C for cooling and 24.4 °C for heating. Hourly estimates of runtimes and air exchange rates were modeled and then summarized to provide annual averages. Values for *λ_HVAC_* were based on air-conditioning system sizing and held constant at 400 cfm per ton of cooling (divided by house volume) for each home type and location combination (full details are provided in Azimi *et al.*, 2015 [64]). HVAC airflow rates were assumed to be the same during both cooling and heating operation and held constant for all filter scenarios. We also assume that filters are replaced on a regular basis and their PM_2.5_ removal efficiencies do not deviate from those shown in Figure 1. We assumed that air exchange rates and HVAC runtimes were the same for all three types of new homes with mechanical ventilation systems. These same inputs varied widely for the infiltration-only homes based on vintage, which fundamentally impacts envelope airtightness, heating and cooling system sizing, and HVAC system runtimes based on differences in heating and cooling loads. In all scenarios, a kitchen range hood with an airflow rate of 100 cfm (170 m^3^/h) was assumed to operate one hour per day along with two bathroom fans (each with an airflow rate of 50 cfm or 85 m^3^/h). 

#### 2.2.7. Estimating the Effectiveness of HVAC Filtration for Reducing Indoor PM_2.5_ of Outdoor Origin

Once annual-average indoor concentrations of PM_2.5_ of outdoor origin (*C_in_*) were estimated for each home type, ventilation strategy, home location, and HVAC filtration scenario, we calculated the effectiveness in reducing PM_2.5_ for each HVAC filter (*E_j_*) by subtracting the ratio of the annual average hourly indoor PM_2.5_ concentration with the filter in question installed (MERV*_j_*) to the annual average hourly indoor PM_2.5_ concentration when only a MERV 5 filter was installed from unity, as shown in Equation (14). MERV 5 was chosen as a baseline for comparison in part because previous work has estimated that approximately 25% of the U.S. residential building stock utilizes filters with a MERV classification of 5 or less [65].

(14) $$E_{MERVj}=1-\frac{C_{in,MERVj}}{C_{in,MERV5}}$$

where:

*E_MERVj_* = filtration effectiveness of MERV _j_ filter for PM_2.5_ of outdoor origin
*C_in,MERVj_* = annual average estimate of the indoor concentration of PM_2.5_ when a MERV_j_ filter was installed (μg/m^3^)
*C_in,MERV5_* = annual average estimate of the indoor concentration of PM_2.5_ when a MERV 5 filter was installed (μg/m^3^)

Finally, we estimated the resulting change in annual average indoor concentrations of PM_2.5_ of outdoor origin with each MERV filter (compared to a MERV 5 filter as the baseline) by multiplying the same concentration predicted with a MERV 5 filter installed by the filtration effectiveness, as shown in Equation (15). Values of Δ*C_in_* for each model scenario were then used in Equation (5) to predict changes in indoor exposures, which were then used in Equations (4) and (8) to estimate the long-term health and economic impacts of HVAC filtration in each home type and location modeled herein.

(15) $$∆C_{in}=E_{MERVj}C_{in,MERV5}$$

## 3. Results and Discussion

Results from the mass balance models and health and economic impact models are shown in the following sections. In total, long-term average indoor concentrations of PM_2.5_ of outdoor origin were estimated for 1452 distinct scenarios spanning 6 home and ventilation system types, 11 HVAC filter scenarios, and 22 geographic locations.

### 3.1. Annual Average Outdoor PM_2.5_ Concentrations in Each Location

Annual average outdoor PM_2.5_ concentrations in each of the 22 U.S. locations are shown in Figure 3. Annual average outdoor PM_2.5_ concentrations ranged from as low as ~5 µg/m^3^ in Pinedale, WY to as high as ~19 µg/m^3^ in Los Angeles, CA, thus capturing a wide variety of outdoor PM_2.5_ concentrations to which the majority of people across the U.S. are exposed. 

**Figure 2:**
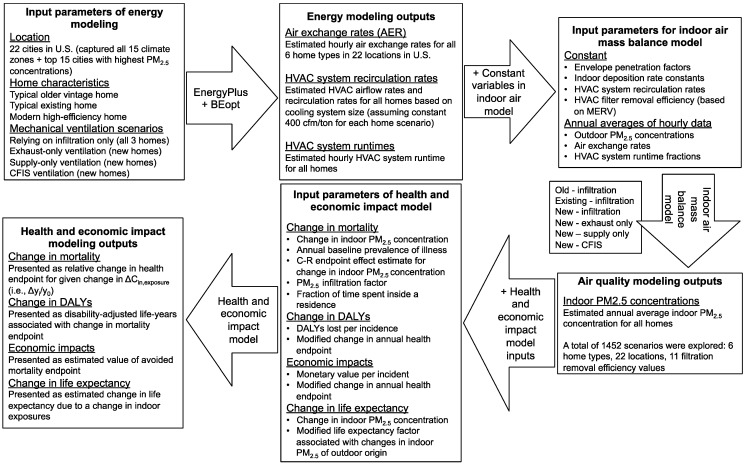
Detailed description of modeling procedures and workflow.

**Figure 3:**
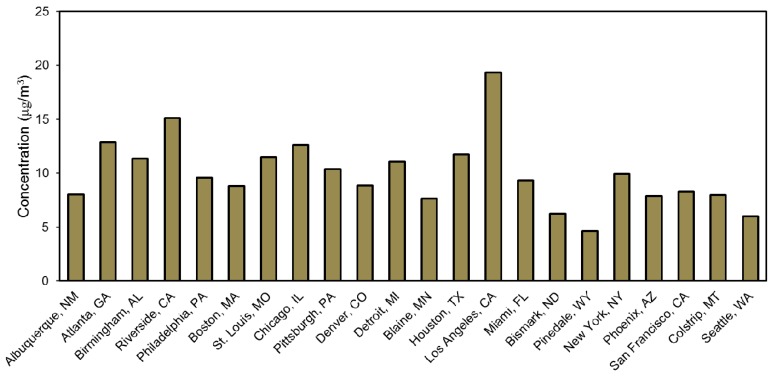
Annual average outdoor PM_2.5_ concentrations for each location [68].

### 3.2. Air Exchange Rates, HVAC System Runtimes, and HVAC Recirculation Rates

**Figure 4:**
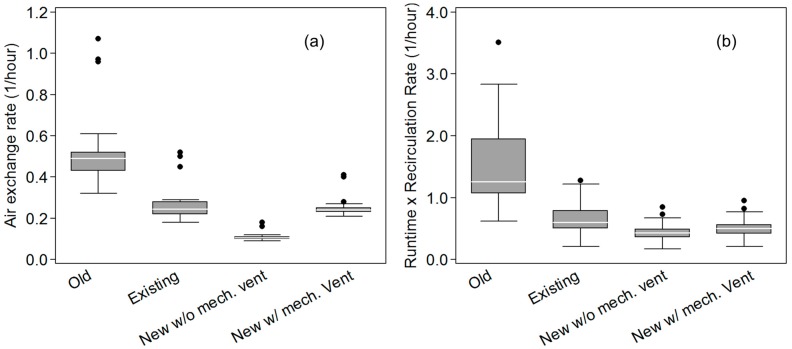
Summary of modeled annual average (**a**) air exchange rates and (**b**) HVAC system recirculation rates multiplied by fractional runtimes (N = 22 locations in each category).

Next, Figure 4a shows distributions of annual average air exchange rates modeled across all 22 locations and the four primary home types (*i.e.*, the three infiltration-only homes including new, old, and existing homes, and the same new home geometry built with mechanical ventilation systems representing all three mechanical ventilation scenarios). Similarly, Figure 4b shows distributions of annual average HVAC system runtimes multiplied by HVAC recirculation rates (*f*×*λ_HVAC_*) for each home type and location.

The median annual average air exchange rate across all locations was just under 0.49/h in the oldest homes, 0.25/h in the existing homes, and 0.11/h in the new homes relying on infiltration alone. The use of constant mechanical ventilation in the new homes increased the median annual average air exchange rate to 0.24/h across all climates. The median annual average HVAC system recirculation rate (multiplied by the average fractional runtime) was 1.26/h for the older homes, 0.60/h for the existing homes, and 0.43/h for the new homes relying on infiltration alone. The addition of constant supply or exhaust mechanical ventilation increased the median value to 0.50/h for the new homes. Differences in home vintages capture differences in both system sizing and runtimes based on varying building envelope characteristics. The median annual average HVAC system runtime across all locations was 20% in the old homes, 13% in the existing homes, and 16% in the new homes relying on infiltration alone. The median annual average runtime increased to 19% in the mechanical ventilation scenarios.

### 3.3. PM_2.5_ Infiltration Factors and Absolute Indoor PM_2.5_ Concentrations

**Figure 5:**
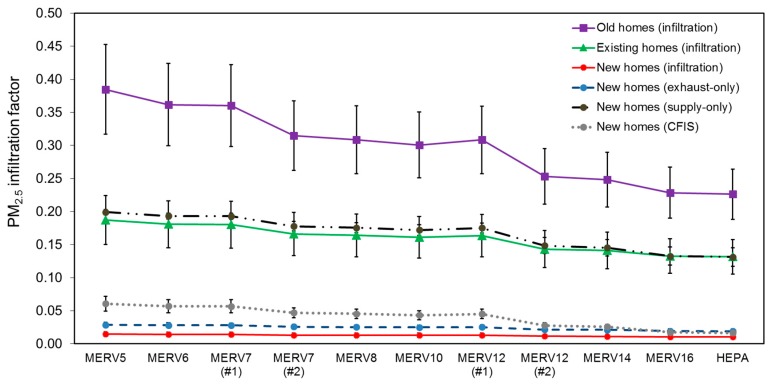
Modeled annual average PM_2.5_ infiltration factors with all 11 HVAC filters installed in each home type and ventilation system combination, averaged across all 22 locations. Modeled home types include old, existing, and new homes relying on infiltration alone, as well as the new home utilizing exhaust-only, supply-only, and central-fan-integrated-supply (CFIS) mechanical ventilation systems.

Figure 5 shows the mean (±S.D.) annual average PM_2.5_ infiltration factors estimated for each of the six home vintage and ventilation system combinations with 11 different HVAC filter scenarios, averaged across all 22 locations. 

Mean (±S.D.) annual average infiltration factors ranged from ~ 0.38 ± 0.07 for the oldest (leakiest) home vintages relying on natural infiltration alone with a low efficiency (MERV 5) filter installed to as little as ~0.01 in the newest home vintages relying on infiltration alone or exhaust-only or CFIS-with-exhaust ventilation with a HEPA filter installed. Resulting infiltration factors for existing (moderate airtightness) homes were similar to the new home with supply-only ventilation, ranging from ~0.2 with a MERV 5 filter installed to ~ 0.15 with a HEPA filter installed. In general, higher efficiency HVAC filtration yielded a decrease in annual average PM_2.5_ infiltration factors in all homes types, with the following classifications yielding similar results to each other: (a) MERV 5, 6, and 7 (#1); (b) MERV 7 (#2), 8, 10, and 12 (#1); and (c) MERV 12 (#2), 14, 16, and HEPA. 

The range of modeled PM_2.5_ infiltration factors (~0.01 to ~0.5) is directly in line with some residential field observations (e.g., [19]) but lower than others (e.g., [9,16]). Differences likely stem from the combination of a restricted number of home types and assumptions considered herein. For example, all of our homes were assumed to have central air-conditioning systems, which tend to yield lower infiltration factors [75]. We also did not account for window opening behaviors, which would have increased infiltration factors [19]. Regardless, we consider these modeled data to provide a reasonable range of PM_2.5_ infiltration factors based on realistic home types and ventilation characteristics for exploration of the likely impacts of HVAC filtration.

Next, we used the same annual average PM_2.5_ infiltration factors to estimate absolute indoor concentrations of PM_2.5_ of outdoor origin in each location for each home type assuming only MERV 5 filters were used, which serves as a baseline for comparison. Annual average indoor concentrations of PM_2.5_ of outdoor origin with a MERV 5 filter installed ranged from approximately 2 to 6 μg/m^3^ in the old homes, from approximately 1 to 3 μg/m^3^ in the existing homes, and from approximately 0.05 to 0.25 μg/m^3^ in the new homes relying on infiltration alone, depending on the annual average outdoor PM_2.5_ concentration, air exchange rate, and HVAC system runtime (multiplied by the recirculation rate) in each location. For the new homes with mechanical ventilation systems, the indoor PM_2.5_ concentration of outdoor origin ranged approximately 0.1 to 0.5 μg/m^3^, 1 to 3.5 μg/m^3^, and 0.3 to 0.8 μg/m^3^ for exhaust-only, supply-only, and CFIS mechanical systems, respectively. Clearly, a wide range of indoor concentrations of outdoor PM_2.5_ exist in residences with low-efficiency HVAC filters installed based primarily on differences in building characteristics, ventilation strategies, and geographic location, which for the homes modeled herein, also drives differences in outdoor PM_2.5_ concentrations, HVAC system runtimes, and HVAC system airflow rates. 

**Figure 6:**
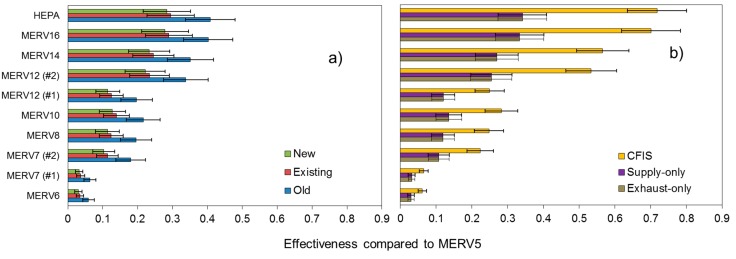
HVAC filtration effectiveness for indoor PM_2.5_ of outdoor origin compared to MERV 5 in (**a**) typical new, existing, and old homes relying on infiltration alone and (**b**) new homes relying on supply-only, exhaust-only, and CFIS mechanical ventilation systems.

Next, Figure 6 shows mean (±S.D.) annual average PM_2.5_ filtration effectiveness values (*E_MERV j_*) for each home type and filter combination compared to the MERV 5 baseline values, averaged across all 22 locations. Figure 6a shows the infiltration-only homes and Figure 6b shows the mechanically ventilated homes. 

Modeled filtration effectiveness ranged from less than 10% to greater than 70% depending on HVAC filter efficiency and the combination of home vintage and type of ventilation system. Relative standard deviations based on geographic location alone were typically 10–20%, suggesting only a moderate influence of location (which influences air exchange rates, HVAC recirculation rates, and HVAC system runtimes in the models). Higher efficiency HVAC filtration had the largest effectiveness for reducing indoor PM_2.5_ of outdoor origin in the new home with a CFIS ventilation system, which is somewhat intuitive given that outdoor air passes directly through the HVAC filter when the HVAC system is operating. The next highest effectiveness of higher efficiency filtration occurred in the older/leakier homes relying on infiltration alone, which is again somewhat intuitive given their higher air exchange rates and HVAC system runtimes. Higher efficiency filtration had the lowest effectiveness in the new, airtight homes regardless of whether they were relying on infiltration alone, supply-only, or exhaust-only mechanical ventilation systems. These data clearly demonstrate that differences in geographic location, building characteristics, and ventilation strategies all contribute to the impact that central residential HVAC filtration can have for reducing indoor PM_2.5_ of outdoor origin.

Finally, Table 2 shows the same data from Figure 5 and Figure 6 combined to estimate the annual average indoor concentration of PM_2.5_ of outdoor origin with each filter installed in the six home types, averaged across all 22 locations. These absolute concentrations provide the basis for estimating the impacts of HVAC filtration on premature mortality and life expectancy associated with long-term indoor exposures to PM_2.5_ of outdoor origin (*i.e.*, Δ*C_in_* calculated using Equation (15)).

**Table 2:** Mean (±S.D.) annual average indoor PM_2.5_ concentrations of outdoor origin across all homes, averaged across all 22 locations (µg/m^3^).

	Old homes	Existing homes	New homes			
	Infiltration-only	Infiltration-only	Infiltration-only	Exhaust-only	Supply-only	CFIS
**MERV 5**	3.70 (± 0.87)	1.80 (± 0.44)	0.15 (± 0.04)	0.28 (± 0.09)	1.99 (± 0.65)	0.58 (± 0.14)
**MERV 6**	3.49 (± 0.85)	1.74 (± 0.43)	0.14 (± 0.04)	0.28 (± 0.08)	1.93 (± 0.64)	0.55 (± 0.13)
**MERV 7 (#1)**	3.47 (± 0.85)	1.73 (± 0.43)	0.14 (± 0.04)	0.28 (± 0.08)	1.93 (± 0.64)	0.55 (± 0.13)
**MERV 7 (#2)**	3.04 (± 0.81)	1.60 (± 0.42)	0.13 (± 0.04)	0.26 (± 0.08)	1.78 (± 0.62)	0.46 (± 0.11)
**MERV 8**	2.99 (± 0.80)	1.58 (± 0.41)	0.13 (± 0.04)	0.25 (± 0.08)	1.76 (± 0.62)	0.44 (± 0.11)
**MERV 10**	2.91 (± 0.79)	1.55 (± 0.41)	0.13 (± 0.04)	0.25 (± 0.08)	1.73 (± 0.61)	0.42 (± 0.11)
**MERV 12 (#1)**	2.99 (± 0.80)	1.58 (± 0.41)	0.13 (± 0.04)	0.25 (± 0.08)	1.76 (± 0.62)	0.44 (± 0.11)
**MERV 12 (#2)**	2.47 (± 0.75)	1.38 (± 0.40)	0.12 (± 0.04)	0.21 (± 0.08)	1.50 (± 0.58)	0.27 (± 0.09)
**MERV 14**	2.42 (± 0.75)	1.36 (± 0.39)	0.11 (± 0.04)	0.21 (± 0.08)	1.47 (± 0.57)	0.26 (± 0.08)
**MERV 16**	2.24 (± 0.73)	1.29 (± 0.39)	0.11 (± 0.04)	0.19 (± 0.07)	1.35 (± 0.55)	0.18 (± 0.07)
**HEPA**	2.21 (± 0.72)	1.28 (± 0.39)	0.11 (± 0.04)	0.19 (± 0.07)	1.33 (± 0.55)	0.17 (± 0.07)

### 3.4. The Impact of HVAC Filters on Premature Mortality Associated with Indoor PM_2.5_ of Outdoor Origin

**Figure 7:**
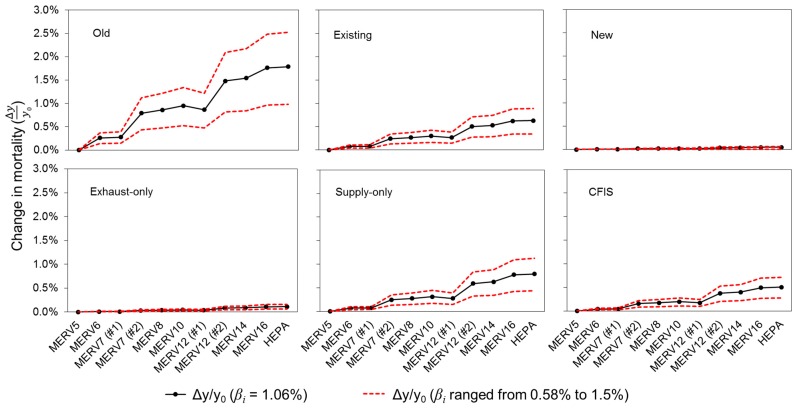
Estimated reduction in premature mortality (Δ*y_i_*/*y*_0_, %) with higher efficiency HVAC filtration in the six primary home vintage and ventilation system combinations, averaged across all 22 locations. Units for *β_i_* are in % per µg/m^3^.

Figure 7 shows the mean estimated reduction in premature mortality associated with long-term exposure to indoor PM_2.5_ of outdoor origin due to higher efficiency HVAC filters in each home type, presented on a relative basis (*i.e.*, Δ*y_i_*/*y*_0_) and averaged across all 22 locations. Solid lines show the mean reductions in premature mortality estimated using a value for *β_i,indoor_* that is based on the mean value of *β_i_* summarized in Section 2.1.2 (*i.e*., *β_i_* = 1.06% per µg/m^3^). Dashed lines show the sensitivity of this estimate to the lower and upper bounds for *β_i_* of 0.58% per µg/m^3^ and 1.5% per µg/m^3^, respectively.

Results from Figure 7 demonstrate that the likely reductions in premature mortality due to higher efficiency HVAC filtration are highest in the oldest/leakiest home types and lowest in the newest home types relying on infiltration alone, which is consistent with large differences in the absolute indoor concentrations of PM_2.5_ of outdoor origin in each home type. The mean predicted reduction in premature mortality compared to MERV 5 filters is as high as ~ 1.6% when using MERV 16 or HEPA filters in the oldest homes, with the selection of *β_i_* yielding a lower bound of ~ 0.8% and an upper bound of ~2.5%. In the same older homes, the use of a MERV 10 filter is estimated to yield an average reduction in premature mortality of ~ 0.4–1.2% (best estimate of ~0.8%), while increasing to a MERV 14 is expected to yield reductions in premature mortality that are only slightly lower than MERV 16 or HEPA. The mean reduction in premature mortality decreases to approximately 0.3–0.7% (best estimate of ~ 0.5%) when using MERV 16 or HEPA filters in the existing home types. The impact of all filters on reducing premature mortality is predicted to be less than 0.1% in the new homes relying on infiltration alone due to minimal outdoor PM_2.5_ penetration into the indoor environment.

Of the three mechanical ventilation scenarios applied to the new homes, the impact of higher-efficiency central HVAC filtration on premature mortality is predicted to be highest with the supply-only systems (~0.4–1.0% with a best estimate of ~0.7%), which is intuitive given that this system supplies outdoor PM_2.5_ directly through their assumed low efficiency filters (*i.e.*, MERV 5). The impact is somewhat lower in the CFIS systems and lowest in exhaust-only systems (which yields results very similar to infiltration-only scenarios). These results demonstrate that the impact of higher efficiency HVAC filtration on premature mortality associated with indoor exposures to PM_2.5_ of outdoor origin varies widely depending on several key assumptions for building characteristics, ventilation strategies, and health effect endpoint estimates. 

### 3.5. Impact of Home Location and Outdoor PM_2.5_ Concentrations on Premature Mortality, DALYs, and Monetized Benefits

**Figure 8:**
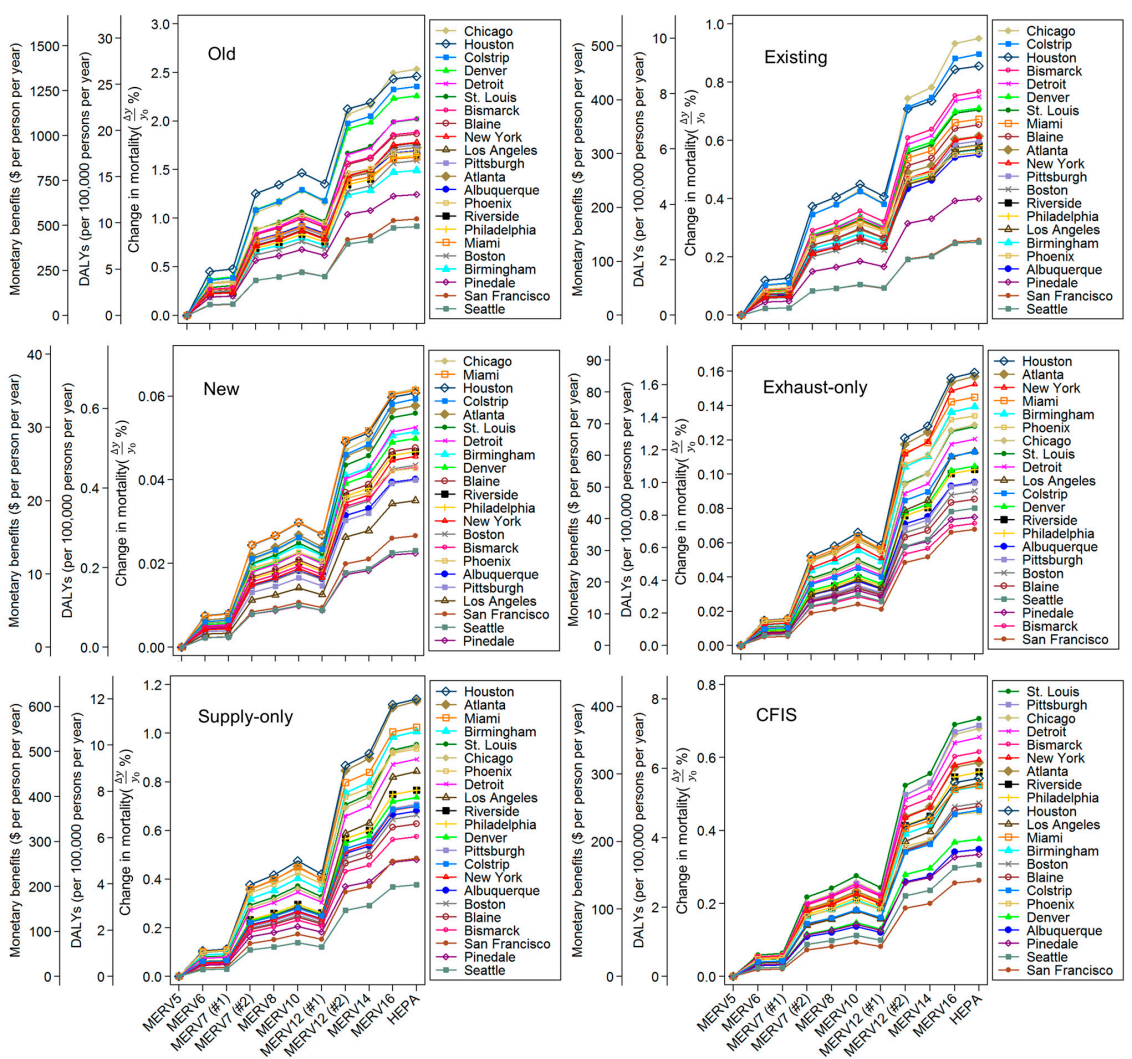
Range of estimated reductions in premature mortality (%), DALYs lost (per 100,000 persons per year), and increased monetary benefits ($ per person per year) for all 11 HVAC filter classifications, six home vintage and ventilation system combinations, and 22 U.S. locations. Note that the graphs are not presented on the same scales for clarity.

Next, Figure 8 combines the estimated reductions in premature mortality (Δ*y_i_*/*y*_0_) with estimates of reductions in DALYs lost (per 100,000 persons per year) and increased monetary benefits ($ per person per year) likely for all 11 HVAC filters modeled in all six home and ventilation combinations, presented separately for all 22 locations in order to explore the influence of geographic location. Only the central estimates of the premature mortality endpoint effects are used for simplicity (*i.e.*, *β_i_* = 1.06% per µg/m^3^, $\frac{\partial DALYs}{\partial disease\;incidence}$ = 1.4, VSL = $7.2 million, and y_0_ = 7.38 × 10^–3^). Note that the y-axes are presented on different scales. Also note that these calculations do not assume any information about population demographics in the homes; they are presented on a per person basis using population-average inputs surveyed in Section 2.1.

Results in Figure 8 demonstrate similar patterns for reductions in premature mortality and DALYs and increases in monetary benefits predicted due to the use of higher efficiency HVAC filters compared to MERV 5 filters in all homes and locations, albeit with large differences in magnitude across home vintages and ventilation system types (as well as smaller differences in magnitude across geographic locations). In general, the predicted health and economic impacts of HVAC filters were similar among the following MERV classifications installed within a given home type: (a) MERV 5, 6, and 7 (#1); (b) MERV 7 (#2), 8, 10, and 12 (#1); and (c) MERV 12 (#2), 14, 16, and HEPA.

The largest reductions in premature mortality and DALYs lost, as well as the largest increases in monetary benefits, likely achievable by higher efficiency HVAC filtration were predicted in the oldest (leakiest) homes relying on infiltration alone. The predicted reduction in premature mortality in the old homes ranged from less than ~ 0.15% for MERV 6 filters installed in Seattle, WA to as high as ~ 2.5% for HEPA filters installed in Chicago, IL. Similarly, the estimated reduction in DALYs lost ranged from ~ 2 per 100,000 persons per year for MERV 6 filters installed in Seattle, WA to ~ 26 per 100,000 persons per year for HEPA filters installed Chicago, IL. The predicted monetary benefits of these same reductions demonstrated the same trends, ranging from a minimum of ~ $58 per person per year for MERV 6 filters in Seattle, WA to a maximum of ~ $1348 for HEPA filters in Chicago, IL.

In the existing homes relying on infiltration alone, the predicted reduction in premature mortality achievable with higher efficiency filters ranged from ~ 0.02% for MERV 6 filters in Seattle, WA to as high as ~ 0.95% for HEPA filters installed in Chicago, IL. The predicted reduction in DALYs lost (and increases in associated monetary benefits) ranged from ~ 0.3 DALYs per 100,000 persons per year (~ $13 per person per year) for MERV 6 filters in Seattle, WA to ~ 10 DALYs per 100,000 persons per year (~ $505 per person per year) for HEPA filters in Chicago, IL. The impacts of HVAC filtration were generally an order of magnitude lower in the new homes relying on infiltration alone, with the predicted reduction in premature mortality with higher efficiency filters ranging from ~ 0.002% for MERV 6 filters in Seattle, WA to ~ 0.06% for HEPA filters installed in Chicago, IL. Corresponding reductions in DALYs lost (and increases in monetary benefits) ranged from ~ 0.03 DALYs per 100,000 person per year (~ $1 per person per year) for MERV 6 filters in Seattle, WA or Pinedale, WY to ~ 0.7 per 100,000 persons per year (~ $33 per person per year) for HEPA filters in Chicago, IL.

The impacts of higher efficiency HVAC filtration on premature mortality in the new homes with exhaust-only ventilation systems were most similar to, albeit slightly greater than, the new homes relying on infiltration alone. The impacts of higher efficiency HVAC filtration in the new homes with either CFIS or supply-only ventilation systems were most similar to the existing homes relying on infiltration alone. For example, the predicted reduction in premature mortality due to higher efficiency filters installed in new homes with exhaust-only systems ranged from less than 0.005% for MERV 6 filters in San Francisco, CA to as high as ~0.16% for HEPA filters in Houston, TX. The corresponding decrease in DALYs lost ranged from ~0.05 DALYs per 100,000 persons per year for MERV 6 filters in San Francisco to ~1.7 DALYs per 100,000 persons for HEPA filters in Houston with corresponding predicted monetary benefits ranging from ~$3 per person per year for MERV 6 filters in San Francisco to ~$85 per person per year for HEPA filters in Houston. 

In the new homes with supply-only ventilation systems, the predicted reduction in premature mortality ranged from ~ 0.029% for MERV 6 filters in Seattle, WA (with a decrease in DALYs lost of ~0.3 per 100,000 persons per year and a monetary benefit of ~$15 per person per year) to as high as ~1.14% for HEPA filters in Houston, TX (with a decrease in DALYs lost of ~12 per 100,000 persons per year and a monetary benefit of ~$607 per person per year). Similarly, in the new homes with CFIS ventilation systems, the predicted reduction in premature mortality ranged from ~0.019% for MERV 6 filters in San Francisco, CA (with a decrease in DALYs lost of ~0.2 per 100,000 persons per year and a monetary benefit of ~$10 per person per year) to ~ 0.7% for HEPA filters in St. Louis, MO (with a decrease in DALYs lost of 7.3 per 100,000 persons per year and a monetary benefit of ~$375 per person per year). 

Combined, these results demonstrate that higher efficiency HVAC filtration can have positive impacts on reducing premature mortality and the associated monetary costs in all of the homes and locations modeled herein. However, there are large differences in the magnitude of impacts achievable with higher efficiency HVAC filtration, ranging from as little as $1 per person per year to as much as $1348 per person per year, based largely on differences in rated HVAC filter efficiency (increasing from MERV 6 to HEPA) and building and ventilation system characteristics that govern particle infiltration. Geographic location, which drives differences in both outdoor PM_2.5_ concentrations and building operational parameters that in part govern removal by central HVAC filtration, had a smaller influence. Although these estimates are all made using central estimates of key input parameters including *β_i_*, $\frac{\partial DALYs}{\partial disease\;incidence}$, $*_i_*, and *y*_0_, many of these parameters have relative uncertainties as high as an order of magnitude that should be taken into consideration when evaluating the absolute magnitude of likely impacts. However, the selection of these input parameters does not drastically impact the relative patterns of health and economic impact estimates for improved HVAC filtration.

### 3.6. Impact of HVAC Filtration on Life Expectancy

**Figure 9:**
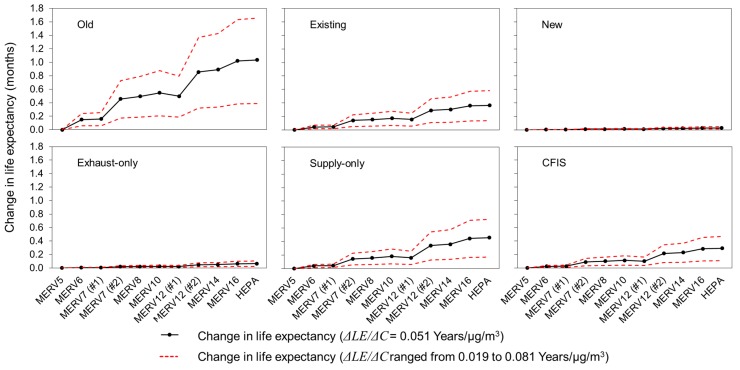
Predicted impacts of higher efficiency HVAC filtration on changes in life expectancy for six combinations of home vintages and mechanical ventilation systems, averaged over 22 locations.

Finally, Figure 9 shows estimates of increases in a similar health endpoint, life expectancy, likely achievable by the use of higher efficiency HVAC filters for all combinations of homes and mechanical ventilation systems, averaged over all 22 locations. The solid lines show an arithmetic mean value of ${\left(\frac{\text{Δ}LE}{\text{Δ}C}\right)}_{outdoor}$ of 0.051 years per µg/m^3^ and the dashed lines show upper and lower bounds of 0.019 and 0.081 years per µg/m^3^. Again, these values are primarily meant to demonstrate an alternative measure of mortality outcomes and should not be counted in addition to changes in premature mortality.

The average increase in life expectancy predicted through the use higher efficiency HVAC filtration (compared to MERV 5 filters) was again greatest for the oldest (leakiest) homes and least for the new homes relying on infiltration alone. The predicted average increase in life expectancy in the oldest homes (averaged across all 22 locations) was predicted to range from ~0.14 months (with upper and lower bounds of 0.24 to 0.06 months) with MERV 6 filters installed to ~1 month (with upper and lower bounds of 1.66 to 0.38 months) with HEPA filters installed. These values decreased to under ~0.05 and ~0.11 months for all MERV classifications installed in the new homes relying on infiltration alone and the new homes with exhaust-only ventilation systems, respectively. The average increases in life expectancy for the existing homes, new homes with supply-only ventilation systems, and new homes with CFIS ventilation systems were similar, ranging from ~0.02–0.04 months for MERV 6 filters to ~0.29–0.46 months for HEPA filters. Again, these results demonstrate that higher efficiency HVAC filtration can have positive health impacts in all of the homes and locations modeled herein by increasing life expectancy, but results vary widely depending on a number of key assumptions for health endpoint effect estimates, location, building characteristics, and ventilation strategies.

### 3.7. Limitations

There are several important limitations to this work that must be mentioned. First, our results are limited only to the specific home types, ventilation strategies, assumptions for input parameters, and geographic locations used herein. We do not take into account regional differences in the prevalence of certain building characteristics, population demographics, or susceptibility to PM_2.5_. Thus, this work does not represent a population-specific effort. Instead, we present results on a per person or population-average basis throughout. Further, our results are limited to the most recent year for which outdoor PM_2.5_ concentrations were available in each location (2012). We also limit our analysis specifically to indoor PM_2.5_ of outdoor origin and do not account for indoor sources, which likely underestimate the benefits of HVAC filtration. We also assume that indoor spaces are well mixed and we ignore the impacts of particle resuspension and window- and door-opening behaviors, which may have led to underestimates of exposure. We also rely only on simple time-averaged methods to estimate long-term averages of indoor PM_2.5_ concentrations of outdoor origin in Equations (9) and (11)–(13). For some locations and home types it may be more appropriate to use a dynamic mass balance method to estimate hourly indoor concentrations using hourly inputs for outdoor PM_2.5_ concentrations, air exchange rates, HVAC systems runtimes, and other parameters such as occupancy (e.g., [64]). 

We also only consider mortality and life expectancy as health endpoints associated with PM_2.5_ but do not account for other associated PM_2.5_ morbidity effects. However, we have described a methodology that can be readily adapted to other health endpoints as well as other pollutants. We also made discrete assumptions for key input parameters such as envelope penetration factors, deposition loss rate coefficients, filtration efficiency, HVAC airflow rates, and envelope airtightness. In reality, there is a wide distribution of most of these parameters in homes (*i.e.*, not all old homes have a penetration factor of 1 and not all new homes have a penetration factor of 0.11). Similarly, HVAC airflow rates were assumed to be the same during both cooling and heating operation and were held constant for all filter scenarios. It is common for heating airflow rates to be higher than cooling airflow rates in some locations, particularly in heating-dominated climates. However, our use of discrete assumptions for each case study home primarily serves to illustrate the impacts of these key parameters and the methods described herein can be readily adapted to a broader statistical analysis in future work.

We also do not consider the influence of filter bypass or duct leakage, primarily for simplicity. We do not consider the cost of any upgrades to older HVAC systems that might be required to enable the installation of higher-pressure drop, higher efficiency HVAC filters such as MERV 16 or HEPA. However, avoiding these assumptions allows us to generalize results without specifying particular filtration products or HVAC system components with specific pressure drop and airflow rate impacts. We also held HVAC airflow rates constant for all filter efficiency scenarios, regardless of likely changes in filter pressure drop that could occur in reality. However, we consider this a reasonably appropriate assumption because (1) there are a number of high efficiency filtration products on the market with extended depths that have reduced pressure drops and can maintain airflow rates in most residential systems [30] and (2) airflow rate reductions in many existing residential HVAC systems are typically less than 10–15% for new higher pressure drop filters otherwise [29,76,77] (and even lower for many newer residential HVAC systems [78]). 

Finally, we also do not take into account the upfront costs of HVAC filtration products in the cost analysis, nor do we attempt to account for differences in operational energy consumption that may occur due to the use of higher efficiency filters. As a simple comparison, recent work suggests that the upfront costs of HVAC filters range from ~ $2 per filter for MERV < 5 filters to $150 per filter for HEPA filters, generally increasing with MERV classification (e.g., [79]). Furthermore, the impacts of higher efficiency filters on residential HVAC energy consumption are typically quite small [29,76,77,78]. Moreover, in previous investigations of the health and economic impacts of higher efficiency filters for reducing PM_2.5_ concentrations in commercial buildings where high-pressure drop filters are thought to yield much larger energy penalties, the monetary benefits of improved health outcomes were much greater than any additional upfront, replacement, or operational energy costs [33,34]. 

## 4. Conclusions 

In this work, we have integrated epidemiology and mass balance methods to evaluate the long-term health and economic impacts of HVAC filtration for reducing premature mortality and increasing life expectancy associated with indoor PM_2.5_ of outdoor origin in residences. We evaluated 11 classifications of HVAC filters ranging from MERV 5 to HEPA in six combinations of single-family home vintages and ventilation systems located in 22 U.S. cities. Results demonstrate that higher efficiency HVAC filters are likely to reduce premature mortality, increase life expectancy, and yield monetary benefits in all of the homes and locations investigated herein. Monetary benefits of reduced premature mortality are predicted to range from $1 to $1348 per person per year, depending on the level of HVAC filtration efficiency used and a number of building and ventilation system characteristics that govern particle infiltration and persistence. The greatest benefits are predicted in older homes that are assumed to allow more outdoor PM_2.5_ to infiltrate indoors. In mechanically ventilated homes, HVAC filters are predicted to have the greatest benefits with supply-only ventilation systems installed, followed by homes with CFIS systems. Benefits are small for new homes relying on infiltration alone or on exhaust-only ventilation systems, as the building envelope is assumed to filter the majority of outdoor PM_2.5_. Across all home types and ventilation system combinations, MERV 7–10 filters appear to offer similar benefits for reducing indoor PM_2.5_ of outdoor origin, while MERV 16 and HEPA appear to offer somewhat diminishing returns over MERV 12–14 filters. Geographic location, which primarily influences outdoor PM_2.5_ concentrations, HVAC airflow rates, HVAC system runtimes, and air exchange rates in the homes modeled herein, also contribute to differences in the impact that HVAC filters can have on health outcomes, albeit typically with a smaller influence than filtration efficiency or other building characteristics.
